# Comparative Transcriptome Analysis of Four Prymnesiophyte Algae

**DOI:** 10.1371/journal.pone.0097801

**Published:** 2014-06-13

**Authors:** Amy E. Koid, Zhenfeng Liu, Ramon Terrado, Adriane C. Jones, David A. Caron, Karla B. Heidelberg

**Affiliations:** Department of Biological Sciences, University of Southern California Los Angeles, Los Angeles, California, United States of America; Beijing Institute of Genomics, China

## Abstract

Genomic studies of bacteria, archaea and viruses have provided insights into the microbial world by unveiling potential functional capabilities and molecular pathways. However, the rate of discovery has been slower among microbial eukaryotes, whose genomes are larger and more complex. Transcriptomic approaches provide a cost-effective alternative for examining genetic potential and physiological responses of microbial eukaryotes to environmental stimuli. In this study, we generated and compared the transcriptomes of four globally-distributed, bloom-forming prymnesiophyte algae: *Prymnesium parvum*, *Chrysochromulina brevifilum*, *Chrysochromulina ericina* and *Phaeocystis antarctica*. Our results revealed that the four transcriptomes possess a set of core genes that are similar in number and shared across all four organisms. The functional classifications of these core genes using the euKaryotic Orthologous Genes (KOG) database were also similar among the four study organisms. More broadly, when the frequencies of different cellular and physiological functions were compared with other protists, the species clustered by both phylogeny and nutritional modes. Thus, these clustering patterns provide insight into genomic factors relating to both evolutionary relationships as well as trophic ecology. This paper provides a novel comparative analysis of the transcriptomes of ecologically important and closely related prymnesiophyte protists and advances an emerging field of study that uses transcriptomics to reveal ecology and function in protists.

## Introduction

Genome sequencing of microorganisms has unveiled a wealth of new information regarding the ecology, physiology and interactions of organisms in the environment. In contrast to most bacteria, archaea and viruses, protistan genomes tend to be large (10–200 Mb compared to 1–10 Mb in bacteria) and more complex, factors that obfuscate bioinformatic analyses, and make for a slower rate of assembly, annotation and gene discovery [1]. The lack of well annotated reference genomes also make *de novo* sequence analysis extremely challenging. Consequently, the current repository of sequenced and annotated eukaryotic genomes covers a small portion of microbial eukaryotic diversity, and is biased toward model organisms and parasitic species that cause human diseases [2], [3].

Transcriptomes contain only the transcribed portions of genomes, which simplifies genetic analyses of eukaryotes by removing complex genetic elements of large intergenic regions, introns and repetitive DNA. In protists, the poly-A+ tail of mRNA transcripts can be selected for sequencing, enriching eukaryotic sequences even in a bacterialized, uni-protistan culture. As such, transcriptomes can be used for the molecular study of protists of interest, circumventing difficult issues such as complicated sequence assembly procedures, to interrogate metabolic and cellular processes.

A mixotrophic nutritional mode among some photosynthetic flagellates (defined here as chloroplast-containing protistan species that also possess the ability for phagotrophy) is a geographically and phylogenetically widespread phenomenon among aquatic protists. A growing body of literature indicates that mixotrophy, especially the consumption of bacteria by phototrophic plankton, is a significant ecological strategy in global marine systems [4]–[7]. Mixotrophy may confer a variety of ecological advantages including carbon, macro- or micronutrient acquisition, and/or supplementation of energy generation [8], [9].

Within the broad spectrum of taxa and nutritional strategies that have been reported, the mixotrophic capabilities of prymnesiophyte (haptophyte) algae have been well documented. Molecular surveys and pigment composition analyses have indicated that prymnesiophytes are globally distributed and abundant in both marine and freshwater ecosystems [10]–[14] where they play key roles in nutrient and organic carbon cycling [15], [16]. Among mixotrophic flagellates studied year-round off the coast of Catalan (Mediterranean), for example, prymnesiophytes were found to be the most important phylogenetic group, accounting for on average 40% of total bacterivory by mixotrophs and 9–27% of total bacterivory [12], [17].

The transcriptomes of four prymnesiophyte algae were compared in this study: *Prymnesium parvum*, *Chrysochromulina brevifilum*, *Chrysochromulina ericina* and *Phaeocystis antarctica*. *P. parvum* is a toxin producer that is capable of developing large, monospecific blooms that cause massive fish kills, ecosystem disruption, and significant economic losses [18]. *Chrysochromulina* spp. are also found globally [19], with some species capable of forming blooms and mass mortality events [20]. *P. antarctica* forms colonies of cells that are embedded in a polysaccharide gel matrix. It is a key species in the Southern Ocean, and is capable of forming blooms of up to 10^7^ cells L^−1^ [21]. *P. antarctica* may also play a significant role in global carbon and sulfur cycles [22], [23]. Three of the four species in this study have also been reported to exhibit mixotrophic nutrition. *P. parvum* can ingest bacteria and other protists, and is capable of taking up organic nutrients [24]–[26]. Members of the diverse genus *Chrysochromulina*, including *C. brevifilum* and *C. ericina* have also been reported to ingest prey [27], [28].

The purpose of this study was to compare the transcripts of closely related prymnesiophytes in order to understand commonalities and differences attributable to both taxonomic relatedness and trophic mode. Our analysis revealed a set of core genes that were shared among all four targeted organisms. The frequencies of functional gene categories in these prymnesiophytes were compared to other protistan organisms in publicly available databases, and indicated that species clustered by genomic information based on both phylogeny and nutritional mode.

## Methods

### Culture conditions

*Prymnesium parvum* (clone Texoma1) was isolated from Lake Texoma, Oklahoma, USA, and made clonal and axenic by micropipetting single cells through rinses of sterile medium. *Chrysochromulina brevifilum* (clone UTEX LB985) and *Chrysochromulina ericina* (clone CCMP281) were obtained from UTEX The Culture Collection of Algae and the National Center for Marine Algae and Microbiota, respectively. *Phaeocystis antarctica* was isolated from the Ross Sea, Antarctica by Robert Sanders. *P. antarctica* was collected under permits issued by the US Antarctic Program through the US Department of State for work in Antarctica. *P. parvum* was collected at the University of Oklahoma Biological Station, Lake Texoma. No collection permits were required.The four study organisms were grown in optimal replete medium for each species (Table 1) in 1–2 L volumes in 2800 ml Pyrex glass Fernbach flasks. Irradiance was measured using a QSL-100 sensor with QSP-170 deckbox (Biospherical Instruments Inc.) and was approximately 300 µE·m^−2^ s^−1^. Cultures were grown in a 12:12 hour light:dark cycle with illumination provided by Philips F20T12CW bulbs. *P. parvum* was grown axenically while the other three species were uniprotistan but non-axenic (bacterized) cultures.

**Table 1:** Culture conditions for the four prymnesiophyte species in this study.

Species	Media	Temp	L:D cycle	Irradiance
*Prymnesium parvum*	L1 –silica, 18 ppt^a^	18°C	12:12	300 µE·m^−2^ s^−1^
*Chrysochromulina brevifilum*	Modified F/2^b^, 30 ppt	18°C	12:12	300 µE·m^−2^ s^−1^
*Chrysochromulina ericina*	LKS^c^ –silica, 36 ppt	18°C	12:12	300 µE·m^−2^ s^−1^
*Phaeocystis antarctica*	LKS^c^ –silica, 36 ppt	1°C	12:12	300 µE·m^−2^ s^−1^

a Salinity is indicated as parts per thousand (ppt).

b Modified F/2 contains the following: NaNO_3_ 2.33 mM; Na_2_HPO_4_ 0.067 mM; No silica; Soil extract; L1 Trace Metals; F/2 vitamins.

c LKS media is a combination of L1 and K media and soil extract (https://ncma.bigelow.org/algal-recipes).

### RNA Isolation

All cultures were harvested during mid-exponential growth phase by centrifugation in an Eppendorf 5810R centrifuge using the A-4-62 rotor at 3200 rcf for 15 min at 15°C. The supernatant was carefully decanted, and 1–2 ml of TRI reagent (Ambion) was added to the pellet and vortexed until the pellet fully dissolved. Homogenates were then either processed immediately using Ribopure kit (Ambion), or stored at −80°C for later processing. The eluted RNA was treated with DNase (Sigma) to remove DNA contamination. Samples were cleaned and concentrated using RNA Clean and Concentrator-25 (Zymo Research). The RNA was quantified using a Qubit 2.0 Fluorometer (Invitrogen) and nucleic acid quality was checked using an E-gel Gel EX 1% (Invitrogen).

### Library Preparation and Sequencing

All samples were quantified again at the sequencing center using Invitrogen Qubit Q32855 and RNA quality was assessed using the Agilent 2100 Bioanalyzer. Libraries were made from 2 µg RNA using Illumina's TruSeq RNA Sample Preparation Kit. The average insert size of each library ranged from 250 to 350 bp. Libraries were sequenced on an Illumina HiSeq 2000 to obtain 2×50 bp (paired-end) reads. Over 2 Gbp of sequence was generated per library. Library preparation and sequencing were performed as part of the Marine Microbial Eukaryote Transcriptome Sequencing Project (MMETSP) supported by the Gordon and Betty Moore Foundation (http://marinemicroeukaryotes.org/).

### Transcriptome Assembly

Transcriptome assemblies for *P. parvum*, *C. ericina* and *P. antarctica* were obtained using the National Center for Genome Research's (NCGR) internal pipeline BPA1.0 (Batch Parallel Assembly version 1.0). Sequence reads were preprocessed using SGA preprocess [29] for quality trimming (swinging average) at Q15. Reads less than 25 nt after trimming were discarded. Preprocessed sequence reads were assembled into contigs with ABySS v.1.3.0 [30], using 20 unique kmers between k = 26 and k = 50. ABySS was run requiring a minimum kmer coverage of 5, bubble popping at >0.9 branch identity with the scaffolding flag enabled to maintain contiguity for divergent branching. Paired-end scaffolding was performed on each kmer. Sequence read pairing information was used in GapCloser v.1.10 [31] (part of SOAP *de novo* package) to walk in on gaps created during scaffolding in each individual kmer assembly. Contigs from all gap-closed kmer assemblies were combined. The OLC (overlap layout consensus) assembler miraEST [32] was used to identify minimum 100 bp overlaps between the contigs and to assemble larger contigs, while collapsing redundancies. The Burrows-Wheeler Alignment (BWA) [33] was used to align sequence reads back to the contigs. Alignments were processed by SAMtools mpileup (http://samtools.sourceforge.net) to generate consensus nucleotide calls at positions where IUPAC bases were introduced by miraEST [32], and read composition showed a predominance of a single base. The consensus contigs were filtered at a minimum length of 150 nt to produce the final set of contigs.

Transcriptome assembly for *C. brevifilum* was obtained using NCGR's internal pipeline BPA2.0 (Batch Parallel Assembly v.2.0). The differences between BPA1.0 and BPA2.0 are as follows: preprocessed sequence reads were assembled into contigs with a newer version of ABySS [30] v.1.3.3, with the scaffolding flag disabled to avoid over-reduction of divergent regions. Unitigs from all kmer assemblies were combined and redundancies were removed using CD-HIT-EST [34] with a clustering threshold of 0.98 identity. The OLC assembler CAP3 [35] was then used to identify minimum 100 bp overlaps between the resultant contigs and assemble larger sequence. The resulting contigs were paired-end scaffolded using ABySS [30]. Sequence read pairing information was used in GapCloser [31] (part of SOAP *de novo* package) v. 1.10 to walk in on gaps created during scaffolding. Redundant sequences were again removed using CD-HIT-EST [34] at a clustering threshold of 0.98 identity. The consensus contigs were filtered at a minimum length of 150 nt to produce the final set of contigs.

The sequences for the four transcriptomes used in this study have been deposited in CAMERA (Community Cyberinfrastructure for Advanced Microbial Ecology Research and Analysis) with the following accession numbers: CAM_ASM_000151 (*P. parvum*), CAM_ASM_000808 (*C. brevifilum*), CAM_ASM_000453 (*C. ericina*), CAM_ASM_000460 (*P. antarctica*).

### Transcriptome Annotation and Comparison

Non-coding ribosomal RNAs and transfer RNAs were detected using RNAmmer [36] and tRNAscan [37], respectively. Coding nucleotide sequences and corresponding translated peptide sequences were predicted using ESTScan [38], [39] with a Bacillariophyta scoring matrix. Sequence reads were aligned back to the nucleotide motifs of the predicted coding sequences using BWA [33]. Peptide predictions over 30 amino acids in length were annotated. Blastp [40] was used to generate hits against the *UniProtKB/Swiss-Prot database*. Protein sequences were also functionally characterized using HMMER3 [41] against Pfam-A [42], TIGRFAM [43], and SUPERFAMILY [44] databases. Only predicted protein sequences longer than 70 amino acids were used in subsequent analyses. These sequences constitute expressed genes or portions of expressed genes, hereafter referred to as “genes”. Genes were also grouped into gene clusters using orthomcl [45]. The resulting data were used in a comparative analysis of predicted gene clusters that were shared and unique among the four transcriptomes.

### Polyketide synthase analysis

Proteins coding for putative polyketide synthase genes were initially identified by a local BLAST against *Emiliania huxleyi* polyketide synthase sequences obtained from GenBank. Sequences with HMM annotations to polyketide synthases were further identified using the NRPS-PKS tool [46] to identify the PKS domains present. Sequences that contained the ketosynthase (KS) domain were used to construct a maximum likelihood tree with 100 bootstraps using the software MEGA5 [47].

### KOG analysis

Putative protein sequences were functionally annotated using KOG categories by blasting all the sequences against the KOG database [48], [49]. The E-value cutoff for a positive hit was 10^−10^. In addition, we used a bit score ratio cut-off, which was derived by dividing the bit score for a hit by the bit score from a self-self hit. A ratio of 0.2 was determined to be a good cut-off after manually evaluating the quality of a number of representative hits.

Predicted protein sequences of 37 other protistan genomes and transcriptomes were downloaded from the JGI website [50] and the CAMERA website [51], respectively. The distribution of these sequences among KOG categories were obtained, as above, by performing a BLAST search against the KOG database and accepting hits that met the E-value and bit score ratio cut-offs of 10^−10^ and 0.2. The KOG category distribution of each species was obtained by dividing the number of hits in each category by the total number of hits to the KOG database. The relative frequencies of each KOG functional category were used to generate a non-metric multidimensional scaling (NMDS) plot using the statistical package R [52] to compare the overall similarity of the organisms to each other.

A subsequent principal components analysis was performed on the relative frequencies of KOG categories across the 41 protistan datasets to confirm the results from the NMDS analysis and to elucidate how much of the KOG frequency pattern could be explained by the principal axes. Additional statistical analyses were done to investigate the influence of trophic mode versus phylogeny on the observed KOG frequencies. Since data were not normally distributed, the influence of phylogeny and trophic mode was tested independently with a non-parametric Krustal-Wallis test, followed by a Steel-Fligner test if significant differences were observed. All statistical analyses and plots were calculated or generated using XLSTAT (v.2013.06.04, Adinsoft TM).

Statistical analysis were used with two datasets: (1) the original dataset of 41 species, with the exception of the mycetozoan, *Dictyostelium purpureum*, the choanoflagellate *Monosiga brevicollis* and the rhodophyte *Cyanidioschyzon merolae*, which were excluded for statistical reasons (no replicates within the phylogenetic group); and (2) a reduced dataset that included only the stramenopiles and prymnesiophytes. The reduced dataset was used to prevent the strong phylogenetic signal present within the alveolates and chlorophytes from masking a signal based on trophic mode.

## Results

### Overview of the transcriptome

Sequencing of the transcriptomes of the four target prymnesiophyte species generated datasets ranging in size from 35.6 million to 61.0 million reads, resulting in 36.4 to 52.0 Mbp of assembled sequence data (Table 2). The number of assembled contigs for *P. parvum, C. brevifilum, C. ericina and P. antarctica* ranged from 30,986 to 56,193. Approximately 72% to 83% of these contigs were predicted to be protein-coding sequences. Non-coding RNAs such as rRNAs and tRNAs formed a small number of the total contigs (Table S1 in File S1). The transcriptome sizes in our study were comparable to other publicly available transcriptomes sequenced by the Gordon and Betty Moore Foundation MMETSP. The complete pathways for essential metabolic and cellular processes were present in each transcriptome, indicating that the sequencing depth provided good coverage. In addition, the number of ribosomal protein genes present in our datasets (94–101) was similar to the number in *E. huxleyi* (99), another prymnesiophyte alga [53].

**Table 2:** Basic statistics for the four transcriptomes sequenced in this study.

Species	Transcriptome size (Mbp)	No. of reads (million)	No. of contigs	No. of peptides	N50
*P. parvum*	36.4	42.9	30,986	25,579	1612
*C. brevifilum*	35.3	35.6	40,494	29,229	1397
*C. ericina*	52.0	61.0	50,899	40,488	1297
*P. antarctica*	50.6	46.6	56,193	45,611	1384

### Core prymnesiophyte genes

**Figure 1:**
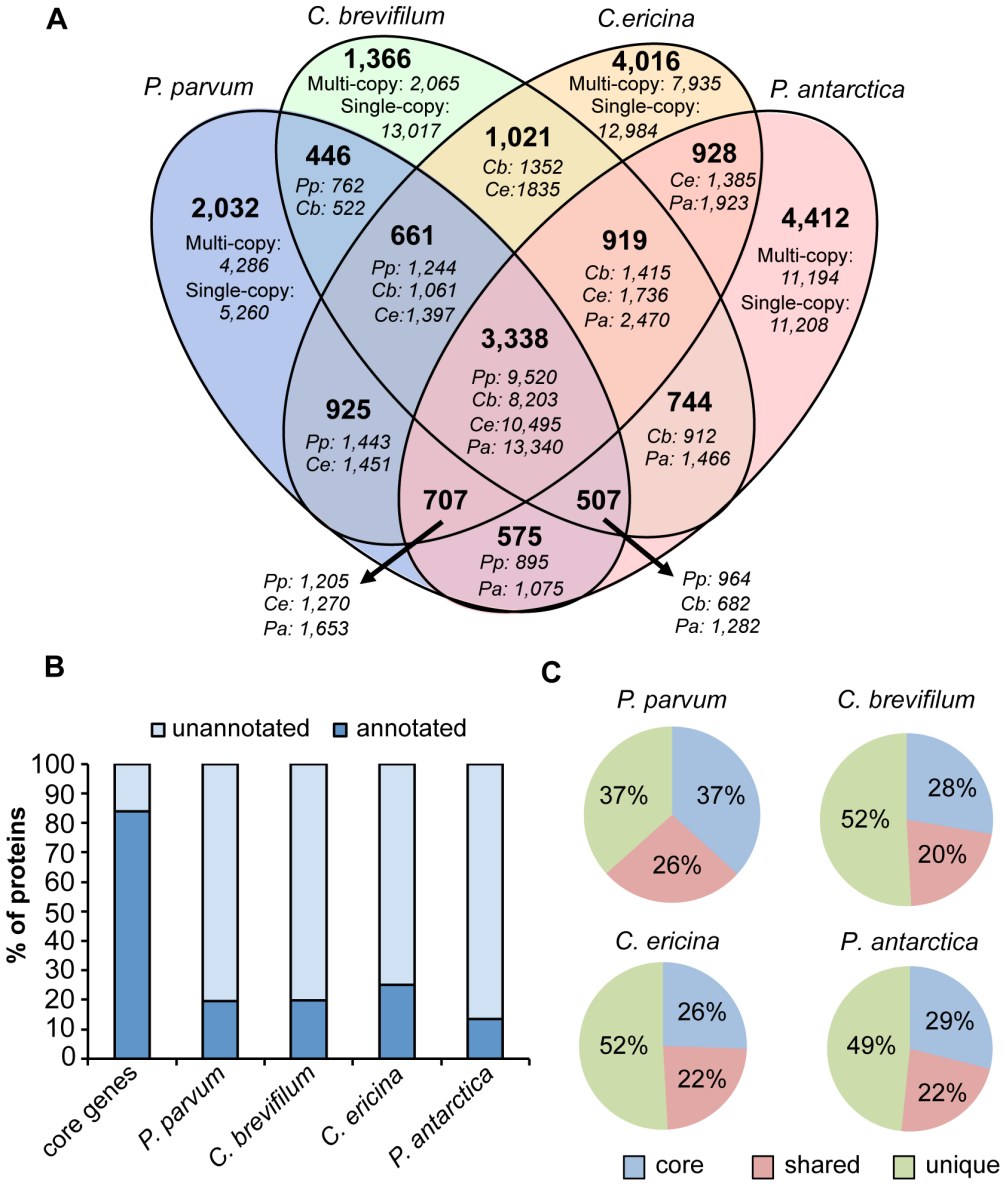
Core, shared and unique transcriptome genes in four prymnesiophyte species: *Prymnesium parvum*, *Chrysochromulina brevifilum*, *Chrysochromulina ericina and Phaeocystis antarctica*. A) Venn diagram showing the number of shared or unique genes (in italics) and gene clusters (in bold) among the four prymnesiophytes as classified by the orthomcl program. Among the genes unique to each of the four prymnesiophytes, multi-copy genes refer to genes that were present in gene clusters, single-copy genes refer to genes that did not cluster with any other gene. Pp: *Prymnesium parvum*, Cb: *Chrysochromulina brevifilum*, Ce: *Chrysochromulina ericina*, Pa: *Phaeocystis antarctica*. B) Proportion of annotated and unannotated genes in the “core” gene set, *i.e.* genes shared by all four species, and in the gene set unique to each species. C) Proportion of the transcripts that comprised core, shared and unique genes. Shared genes are genes present in two or three of the four species. Unique genes are genes that are only present in one species.

We found a set of gene clusters that were common to all four prymnesiophyte species (“core transcriptome”). This core set of proteins consisted of 3,338 gene clusters (Fig. 1A), which comprised 37%, 28%, 26% and 29% of the total predicted proteins in *P. parvum, C. brevifilum, C. ericina and P. antarctica*, respectively (Fig. 1C). *C. brevifilum* and *C. ericina* shared the most gene clusters (1,021) followed by *P. parvum* and *C. ericina* (925) and then *P. antarctica* and *C. ericina* (928) (Fig. 1A). Each transcriptome contained unique gene clusters (each gene cluster was comprised of multi-copy genes) not found in the other three transcriptomes; the number of gene clusters that were unique to each species were 2,032 (*P. parvum*), 1,366 (*C. brevifilum*), 4,016 (*C. ericina*) and 4,412 (*P. antarctica*). In addition to the unique gene clusters, each species also had unique single-copy genes (Fig. 1A). The unique gene clusters and single-copy genes together comprised 37–52% of the transcriptomes (Fig. 1C). *C. brevifilum* and *C. ericina* had the highest proportions of unique genes (each with 52%), while *P. antarctica* had 49% and *P. parvum* had 37% (Fig. 1C). Approximately 20–26% of the transcriptomes was comprised of proteins that were shared between at least two of the four prymnesiophyte species. Of the 3,338 gene clusters shared among all four species, 2,752 (82%) were annotated by KEGG or orthomcl. In contrast, the number of annotated unique genes in each species was much smaller: *P. parvum*: 1,398 (20%); *C. brevifilum* 2,828 (20%); *C. ericina*: 4,205 (25%); *P. antarctica*: 2,057 (13%) (Fig. 1B).

The majority of core prymnesiophyte proteins were mapped by KEGG to essential metabolic pathways such as the biosynthesis of amino acids, carbon metabolism, fatty acid metabolism and purine and pyrimidine metabolism. All or nearly all the genes necessary for these pathways were present. For instance, all of the TCA cycle genes were found in *C. brevifilum*, *C. ericina* and *P. antarctica*, but in *P. parvum*, one enzyme, fumarate dehydratase, was either not recovered or annotated. All of the genes for glycolysis/gluconeogenesis were present in the four transcriptomes. The pathways for the biosynthesis of some amino acids, such as methionine and proline, were missing one or two genes in each transcriptome. The non-mevalonate pathway or 2-*C*-methyl-D-erythritol 4-phosphate/1-deoxy-D-xylulose 5-phosphate pathway (MEP/DOXP pathway) that is part of the terpenoid backbone biosynthesis pathway was also nearly complete. The core transcriptome also included genes for the synthesis of ribosomal proteins, proteasomes and spliceosomes. Pathways with only some of the necessary genes include the biosynthesis of other secondary metabolites, starch and sucrose metabolism, and the urea cycle. Some genes involved in the metabolism of cofactors and vitamins were present in all four species, however, the core transcriptome did not contain the full pathway for the metabolism of any of the cofactors and vitamins.

We also compared the core transcriptome of our four prymnesiophyte species with the predicted proteins from the *E. huxleyi* genome. Of the 1,433 proteins with KEGG annotations that were in our shared core transcriptome, 1,303 (91%) were also present in *E. huxleyi*. There were 130 predicted proteins that were present in *P. parvum*, *C. brevifilum*, *C. ericina* and *P. antarctica* but not in *E. huxleyi*. They consisted of a few enzymes in various pathways, such as nitrogen metabolism (1), cysteine and methionine metabolism (1), and TCA cycle (3), among others. They also included alternative enzymes for the same pathway, or alternative pathways for the same substrate/product conversion. In some cases, a protein that was unique to the four target species might be involved in a different intermediate pathway that feeds into the same main pathway, with no resulting change in the final product. For example, isocitrate dehydrogenase, which is present in the core transcriptome of the four target prymnesiophyte species but not in *E. huxleyi*, can convert isocitrate to oxalosuccinate to 2-oxoglutarate whereas the gene that is present in *E. huxleyi* converts isocitrate directly to 2-oxoglutarate. In both cases, isocitrate is converted to 2-oxoglutarate, which are two major compounds in the TCA cycle.

### B-vitamin biosynthesis pathways

A majority of the predicted proteins unique to each species were not annotated, making it more challenging at this time to understand the differences among the different species. Nonetheless, some of the differences in the proteins were present in three vitamin biosynthesis pathways, specifically thiamine, biotin and cobalamin.

The number of predicted proteins involved in thiamine biosynthesis was variable among the four target species. The *P. parvum* and *P. antarctica* transcriptomes contained only the IscS gene, which is a cysteine desulfurase (Fig. A in File S1). Although this enzyme is part of the thiamine metabolic pathway, it also functions in the sulfur relay system, which may explain its presence in the two species that did not otherwise have other key thiamine synthesis enzymes. In contrast, the two *Chrysochromulina* species, and *C. ericina* especially, possessed an interesting composition of genes that belong to this pathway. The synthesis of thiamine pyrophosphate (TPP)—the biologically active form of thiamine—begins with the formation of a pyrimidine moiety (HMP-PP) and a thiazole moiety (HET-P). The former is formed by THIC and THID from 5-aminoimidazole ribonucleotide (AIR), while the latter is catalyzed by THI4 and THIM from NAD+, glycine and an unknown sulfur-containing compound. In bacteria, HET-P is synthesized *de novo* from 1-deoxy-D-xylulose 5-phosphate (DXP) by a suite of different enzymes [54]. The ThiE enzyme catalyzes the condensation of HMP-PP and HET-P to form thiamine monophosphate. In some organisms, the function of THID and ThiE are combined into one enzyme. Thiamine monophosphate is dephosphorylated by an unknown phosphatase to form thiamine that is then pyrophosphorylated by TPK into thiamine pyrophosphate. *C. ericina* has ThiE and TPK, and as such, given both the pyrimidine moiety and the thiazole moiety, might be able to synthesize TPP. However, *C. brevifilum* had the ThiDE and the ThiE gene but not TPK, and might have a reduced and potentially non-functional thiamine biosynthesis pathway (Fig. A in File S1).

The presence of biotin metabolism genes in the four transcriptomes was also variable. In bacteria, four enzymes convert pimeloyl CoA to biotin in a sequential fashion, beginning with BioF, followed by BioA, BioD and finally, BioB [55]. Genomes of protists that do not need exogenous thiamine such as *Thalassiosira pseudonana*, *Chlamydomonas reinhardtii* and *Cyanidioschyzon merolae* have been found to contain the *bioF*, *bioA* and *bioB* genes (data culled from the KEGG database). However, because these protists are able to synthesize biotin, it has been hypothesized that an unknown enzyme might carry out the activity of the missing BioD enzyme [56]. Regardless, none of our four prymnesiophytes had all three genes. *P. parvum* had *bioF* and *bioA* while *C. ericina* and *P. antarctica* had *bioF* and *bioB*. *C. brevifilum* only had the *bioF* gene.

All four prymnesiophyte species contained the CobW gene sequence, which is annotated as a cobalamin synthesis protein. The numbers of putative CobW genes differ among the four transcriptomes, with the smallest number in *P. parvum* (2 copies) and the largest in *C. ericina* (10 copies) (Table S2 in File S1). In addition, *C. brevifilum* also had a predicted protein with sequence similarity to cobyrinic acid a,c-diamide synthase, or CobB. *C. ericina* and *P. antarctica* both had a CobN-like protein, which is a cobaltochelatase. *P. parvum*, *C. brevifilum* and *C. ericina* all possessed the B_12_-dependent form of methionine synthase MetH, but not the B_12_-independent form, MetE. All four prymnesiophytes also had the methylmalonyl-coA mutase (MCM) gene in their transcriptomes (Table S2 in File S1).

### Polyketide synthase genes

**Figure 2:**
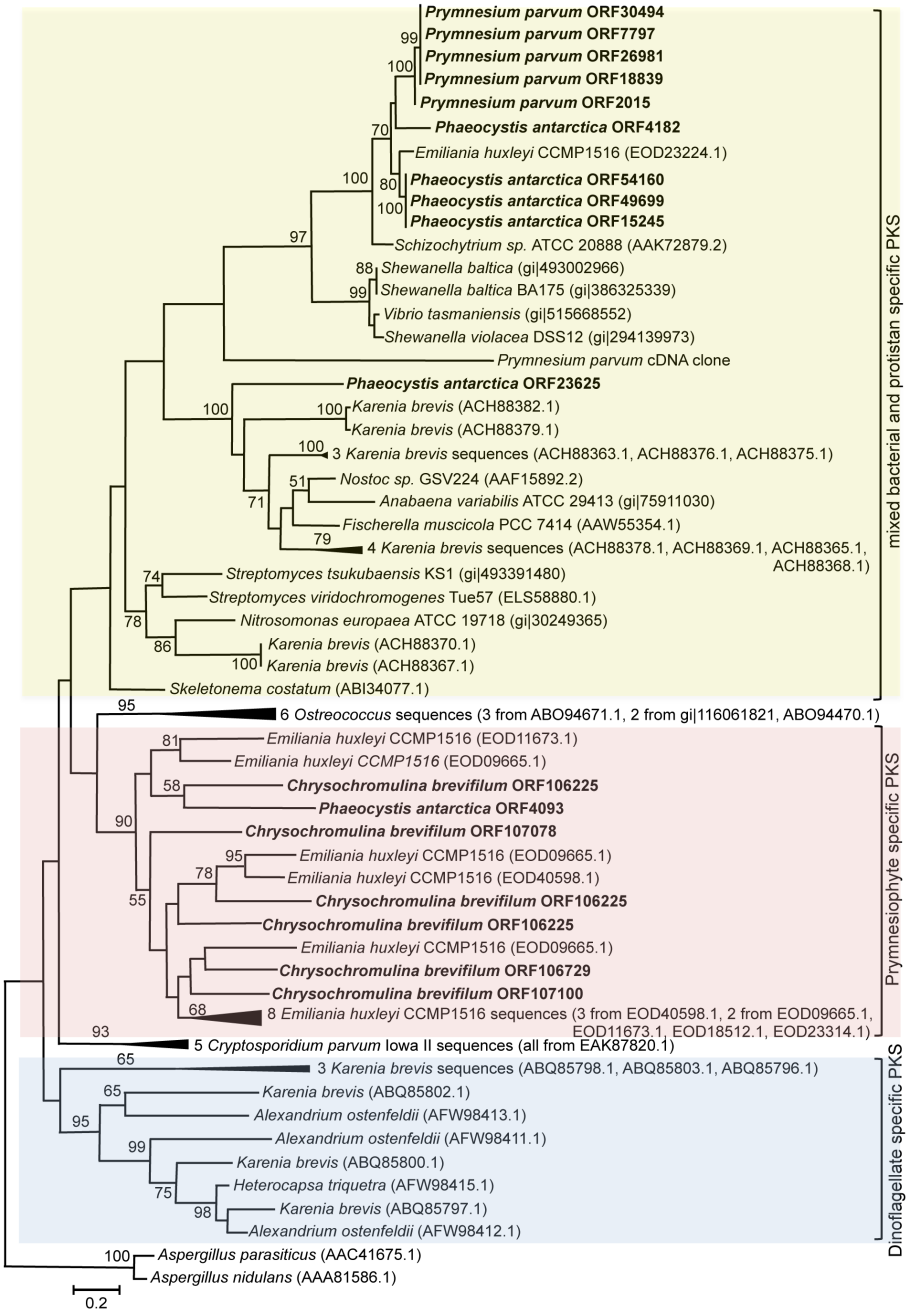
Polyketide synthase maximum likelihood tree with 100 iterated bootstraps using only the keto-synthase (KS) domain. The tree was inferred using MEGA5 (Tamura et al. 2011) with maximum likelihood method based on Jones-Taylor-Thornton model. The analysis involved 78 amino acid sequences. All positions with less than 95% site coverage were eliminated. There were 181 total sites in the final dataset. Bootstrap support values, if greater than 50%, are shown as the percentages of 100 trees inferred in the analysis. The scale bar represents the number of substitutions per site. The tree is rooted with *Aspergillus nidulans* polyketide synthase. Sequences from our dataset are shown in bold. Multiple branches have the same identifying ORFs,GI or accession numbers due to multiple KS domains on the same gene.

**Figure 3:**
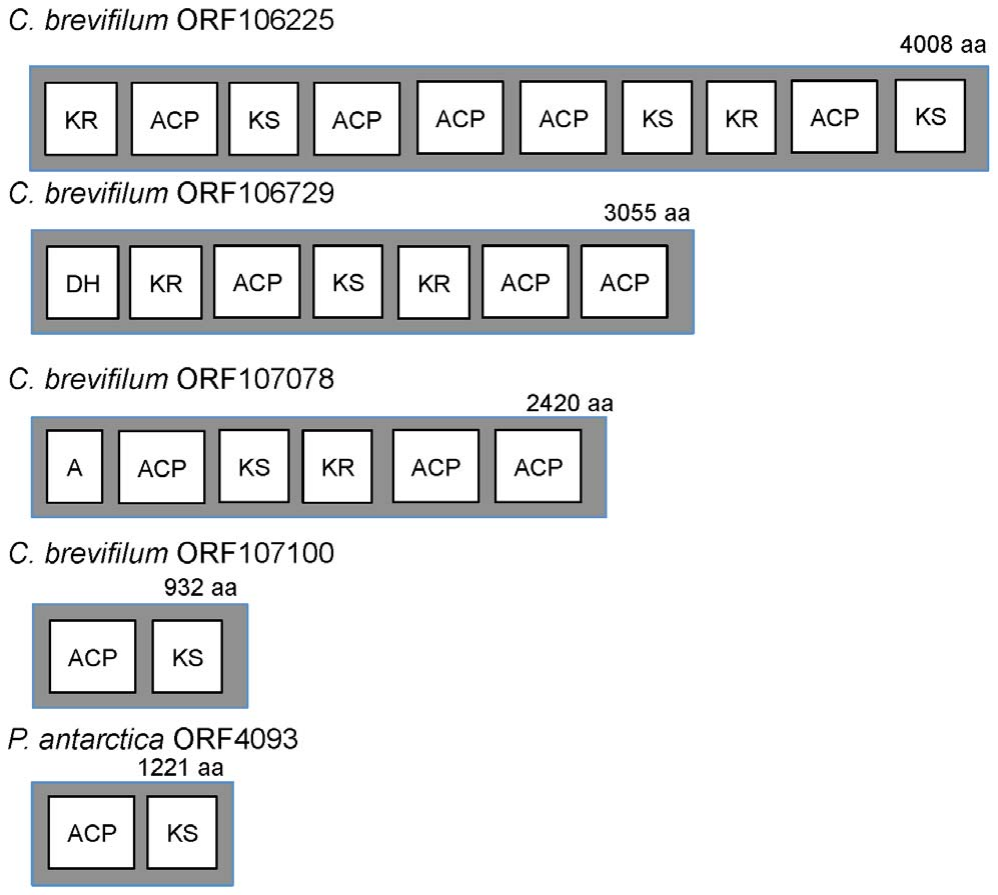
Putative domain organization and length of polyketide synthase sequences in genes containing more than one domain, as annotated by the NRPS-PKS tool. KR: ketoreductase, ACP: acyl carrier protein, KS: keto-synthase, DH: dehydratase, A: adenylation.

Our datasets contained 15 putative polyketide synthase genes with a ketosynthase (KS) domain. These sequences were found in three out of four of the target species: *P. parvum*, *C. brevifilum* and *P. antarctica* (Table S3 in File S1). The phylogeny of our prymnesiophyte KS sequences was analyzed by generating a maximum likelihood tree. This analysis showed that our prymnesiophyte KS sequences fell into two distinct clusters (Fig. 2). All the sequences from *C. brevifilum* and one sequence from *P. antarctica* (ORF4093) clustered with *E. huxleyi* KS sequences in a prymnesiophyte-specific PKS clade. Meanwhile, all the sequences from *P. parvum* and five sequences from *P. antarctica* were dispersed among sequences from a diverse group of species, including *E. huxleyi*, *Karenia brevis* and various bacteria. All the sequences that fell into the prymnesiophyte-specific PKS clade contained multiple PKS domains (Table S3 in File S1) with variable organizations. The longest PKS sequence, ORF106225, contained two ketoreductase (KR), three ketosynthase (KS) and five acyl-carrier protein (ACP) domains (Fig. 3). Another sequence contained the KR, KS and ACP domains as well as a leading dehydratase (DH) domain. A domain that carries out adenylation (A domain) was found at the start of ORF107078, followed by ACP, KS and KR domains (Fig. 3). In *P. antarctica*, ORF4093 contained one ACP domain and one KS domain. In contrast, sequences that clustered with the mixed bacterial/protistan PKS clade only contained the KS domain.

### KOG patterns

**Figure 4:**
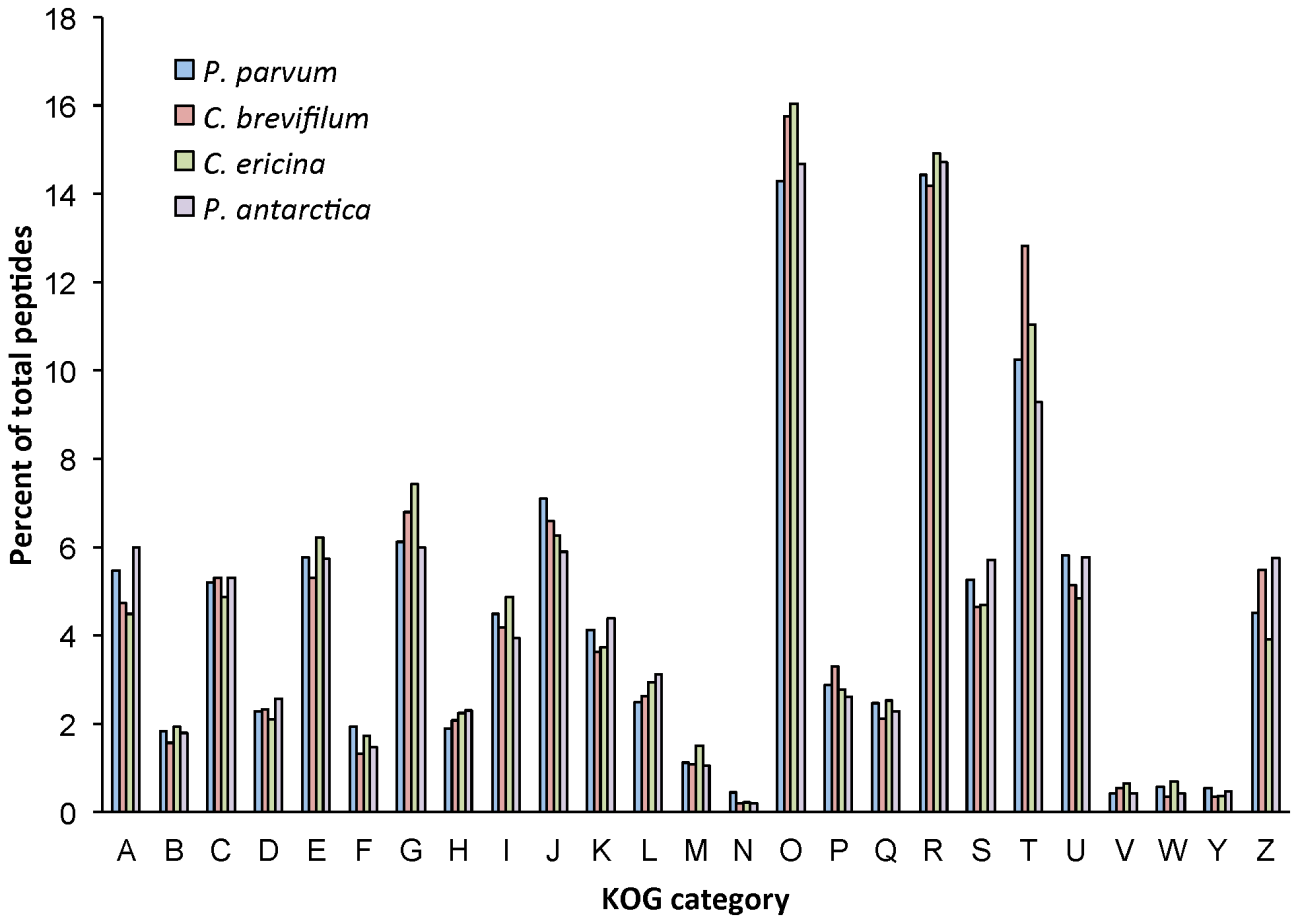
KOG function distribution of the peptides for the four target species in this study. The KOG functions are as follows: A: RNA processing and modification; B: chromatin structure and dynamics; C: Energy production and conversion; D: Cell cycle control, cell division, chromosome partitioning; E: Amino acid transport and metabolism; F: Nucleotide transport and metabolism; G: Carbohydrate transport and metabolism; H: Coenzyme transport and metabolism; I: Lipid transport and metabolism; J: Translation, ribosomal structure and biogenesis; K: Transcription; L: Replication, recombination and repair; M: Cell wall/membrane/envelope biogenesis; N: Cell motility; O: Posttranslational modification, protein turnover, chaperones; P: Inorganic ion transport and metabolism; Q: Secondary metabolites biosynthesis, transport and catabolism; R: General function prediction only; S: Function unknown; T: Signal transduction mechanisms; U: Intracellular trafficking, secretion and vesicular transport; V: Defense mechanisms; W: Extracellular structures; Y: Nuclear structure; Z: Cytoskeleton.

EuKaryotic Orthologous Groups (KOGs) is a tool used to identify orthologous and paralogous proteins in eukaryotes, and assigns a functional category [48]. The overall distribution of KOG functions of the four prymnesiophytes in this study was very similar (Fig. 4). Generally, the KOG category with the greatest number of peptides was O (posttranslational modification, protein turnover, and chaperones). This was closely followed by R (general function prediction only). The greatest difference between any two species was observed for KOG function T, signal transduction mechanisms. *C. brevifilum* had the greatest number of peptides belonging to this category relative to the other species, followed by *C. ericina* (2.1% less than *C. brevifilum*).

**Figure 5:**
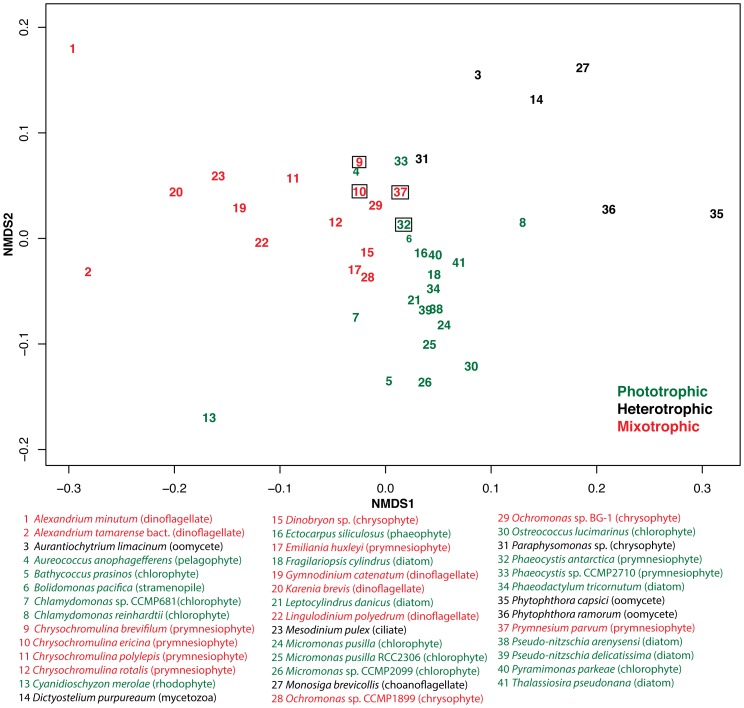
Nonmetric multidimensional scaling (NMDS) plot of the KOG distributions of the four prymnesiophytes in this study and of other protistan genomes and transcriptomes. The genomes were obtained from the Joint Genome Institute database, and the transcriptomes were obtained from the Marine Microbial Environmental Transcriptome Sequencing Project (MMETSP) database. The stress value for this plot was 0.12, which indicates that the two-dimensional plot is a good representation of the data. The four target species in this study are highlighted in boxes. The trophic modes of each organism are denoted in green (phototrophs), black (heterotrophs) and red (mixotrophs).

Comparing the KOG distribution of our four species to that of other protists in an NMDS analysis resulted in distinct groupings that appeared to reflect both phylogenetic relationships and nutritional mode (Fig. 5). The stress value of the NMDS was 0.12, indicating that the 2D plot was a good depiction of the separation among the data. We found that heterotrophic species such as the choanoflagellate, *Monosiga brevicollis*, the stramenopile, *Paraphysomonas imperforata*, the slime mold, *Dictyostelium purpureaum* and water molds, *Phytophthora capsici* and *P. ramorum*, occupied a wide area of the plot, but occupied a space separate from species that are autotrophic or mixotrophic (Fig. 5). The autotrophs formed a group that overlapped somewhat with species that are known to be photosynthetic species capable of phagotrophy (i.e. mixotrophic). The latter group had a large spread, mostly due to the position of the dinoflagellates, which occupied an area distinct from other groups. The prymnesiophytes and chrysophytes clustered together in a region of overlap between non-phagotrophic autotrophic and mixotrophic protists. Some phylogenetic groups formed distinct clusters, such as the diatoms *Pseudo-nitzschia arenysensi*, *Pseudo-nitzschia delicatissima*, *Phaeodactylum tricornutum*, *Fragilariopsis cylindrus* and *Leptocylindrus danicus*. Additionally, the prasinophytes, *Ostreococcus* and *Micromonas*, clustered together. In contrast, the other two chlorophytes, *Chlamydomonas reinhardtii* and *Chlamydomonas* sp. CCMP681 did not cluster with each other, and the chrysophyte congeners, *Ochromonas* sp. CCMP1899 and *Ochromonas* sp. BG-1 also did not group together.

**Figure 6:**
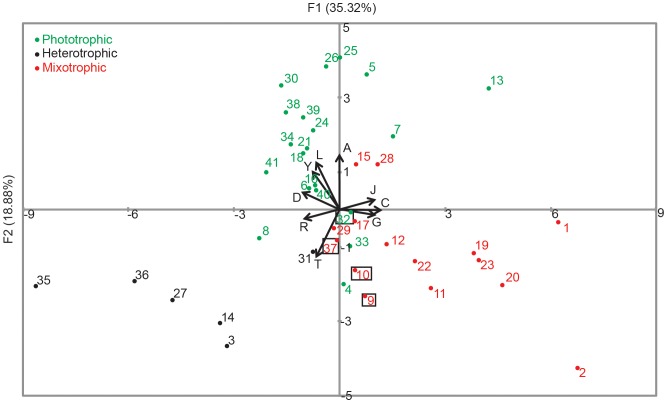
Principal component analysis (PCA) plot of the KOG distributions of the four prymnesiophytes in this study and of other protistan genomes and transcriptomes. The same dataset from Fig. 5 was used to generate this figure. The color scheme and species identification by numbering also correspond to Fig. 5. Explained cumulative variability for this plot was 54.2%, with eigenvalues of 8.5 (F1) and 4.5 (F2). Only top variables for F1 and F2 are plotted in the graph.

We undertook a more detailed principle components analysis (PCA) to confirm and elucidate the NMDS results. The spatial distribution of the PCA plot was in concordance with that of the NMDS (Fig. 6), but the two principle axes accounted for only 54% of the variability, indicating that 46% was not explained by this representation. The top five variables that explained up to 40% of the variability in the first axis were: C (energy production and conversion), D (cell cycle and division), G (carbohydrate transport and metabolism), R (general function prediction only) and J (translation and ribosomal structure). Along the second axis, up to 55% of the variability could be accounted for by the following five variables: A (RNA processing), T (signal transduction mechanisms), L (replication, recombination and repair), Z (cytoskeleton) and Y (nuclear structure).

Statistical analyses performed on each of the KOG functions were able to tease out specific functions that were different based on either phylogeny or trophic mode. In the original large dataset, phylogenetic grouping had a statistically significant effect in ten KOG categories (Table S4 in File S1), while trophic mode was significant for six KOG categories. After removing the alveolates and the chlorophytes, assuming that their strong phylogenetic signal might bias the results, four KOG categories retained significant differences based on trophic mode, namely: D (cell cycle control, cell division and chromosome partitioning), G (carbohydrate transport and metabolism), H (coenzyme transport and metabolism) and K (transcription).

**Figure 7:**
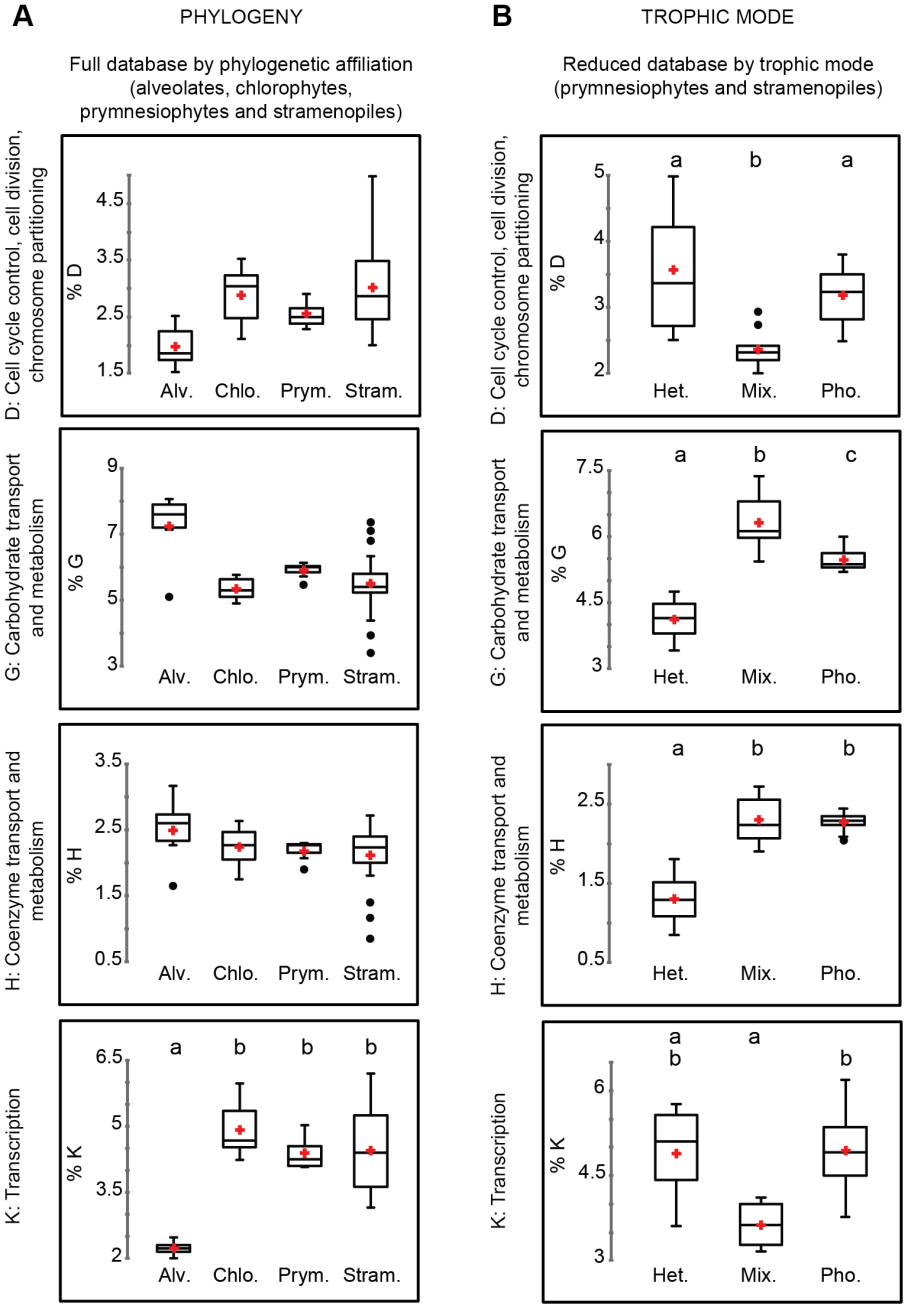
Box plots of the proportion of genes assigned to KOG functions that had a statistically significant difference among phylogenetic and trophic modes (see Table S4). A) The full dataset of 41 species (excluding the mycetozoan *Dictyostelium purpureum*, the choanoflagellate *Monosiga brevicollis* and the rhodophyte *Cyanidioschyon merolae*) showing the proportion of genes annotated with a particular KOG function and grouped by higher-level taxonomic affiliation; B) A reduced dataset of the proportion of genes assigned to particular KOG function in the prymnesiophytes and stramenopiles, grouped by trophic modes. The alveolates and chlorophytes were excluded to reduce phylogenetically-based bias in the dataset. Small case letters over each bar summarize the different statistical groups found by multiple pairwise comparisons. Red crosses indicate the mean for each group and black dots represent the outliers.

Box plots of the frequencies of the different KOG categories showed interesting patterns (Fig. 7). Grouped by phylogenetic relatedness, alveolates generally had KOG functions that differed from chlorophytes, prymnesiophytes and stramenopiles (Fig. 7: column of panels on left). These patterns were different for taxa grouped by nutritional modes (), once the alveolates were removed to reduce their strong effect (Fig. 7: column of panels on right). For example, the frequency of KOG function D (cell cycle control, cell division and chromosome partitioning, top row of panels) was significantly different for mixotrophic species compared to heterotrophs and autotrophs, which were similar to each other (Fig. 7: top right panel). For KOG category G (carbohydrate transport and metabolism, right hand column, panel second from top), all three trophic modes had frequencies that were statistically significant from each other. In category H (coenzyme transport and metabolism, right hand column, panel third from top), the heterotrophic group was significantly different from the mixotrophic and photosynthetic groups. The frequency of KOG function K (Transcription, bottom right panels) was significantly different in the mixotrophs compared to the phototrophs, but not significantly different compared to the heterotrophs. There was no significant difference between the heterotrophs and the phototrophs for this KOG category.

## Discussion

The numbers of predicted peptides for the four species in this study varied greatly, but were within the range of other MMETSP dataset transcriptomes and the number of predicted protein coding genes in *E. huxleyi* (30,569) [53]. The high number of peptides was not due to bacterial contamination because preparation of the cDNA biased against bacterial mRNA, and we observed negligible numbers of bacterial genes in the transcriptomes. It is possible that the large number of peptides in part reflects the existence of fragments of the same gene called as two different genes in cases where the sequence that joins the two fragments was not sequenced. However, the N50 for these four datasets were between 1,297 and 1,612 aa (Table 2), which is close to the average gene length for eukaryotes [57].

### Core, shared and unique genes

Our results indicate that the four prymnesiophytes in our study share a core set of genes that may also be more broadly shared among other prymnesiophytes. These genes code for essential cellular and metabolic functions such as carbon metabolism, amino acid synthesis, DNA synthesis and fatty acid metabolism. The transcriptomes also contained “shared” genes, that were observed in two or three of the four target species. There is limited data on how much physiological diversity might be present among congeneric protistan species because these taxa have been traditionally defined based on morphological features. Therefore, we were interested in what our data might reveal about relatively closely related taxa. The congeners *C. brevifilum* and *C. ericina* shared more gene clusters with each other than either did with *P. parvum* or *P. antarctica*. The proportion of genes shared between these two species (5%) was greater than the proportion of genes *Thalassiosira pseudonanna* (a centric diatom) shared with *Fragilariopsis cylindrus* (3%) and *Pseudo-nitzschia multiseries* (2%) (pennate diatoms), but much lower than the proportion of genes shared between *F. cylindrus* and *P. multiseries* (26–28%) [58].

The remainder of the genes in each of our four prymnesiophyte transcriptomes were only found in a single species (Fig. 1A, C). Approximately half of the transcriptome of *C. brevifilum*, the species with the largest transcriptome in our study, consisted of genes unique to that species. However, this percentage is within the range of other studies. The proportion of unique genes in our four transcriptomes (37–52%) was similar to the results of a study that compared three diatom transcriptomes: *Thalassiosira pseudonana*, *Fragilariopsis cylindrus* and *Pseudo-nitzschia multiseries* (39–43%) [58]. A majority of our unique proteins were not annotated due to the limited genomic databases for free-living, environmentally relevant microeukaryotes. Consequently, the proportion of peptides that were annotated in this study (33% to 47%) was similar to recent results that have been obtained for other sequenced protistan transcriptomes. For example, 41% and 31% of the transcriptomic sequences obtained from two dinoflagellates within the genus *Symbiodinium* were annotated [59], while 33% of the contigs from another dinoflagellate transcriptome, *Heterocapsa circularisquama* were annotated [60]. Only 23% of the transcriptome of the heterotrophic dinoflagellate, *Oxyrrhis marina*, could be annotated using a variety of databases, including Genbank's nr database [61]. The same pattern of a highly annotated core genome compared to poorly annotated unique genes has also been reported previously [62].

### B-vitamin biosynthesis genes

Each of our four prymnesiophytes showed slightly different abilities to synthesize vitamins. This result was not surprising as such differences have been observed within genera and even among different strains of the same species of algae [63].

Thiamine is a cofactor for enzymes involved in many different metabolic pathways, including carbohydrate and amino acid metabolism. Thiamine auxotrophy is widespread among protistan species, with 20% of eukaryotic phytoplankton surveyed requiring exogenous thiamine [56]. The proportion of thiamine auxotrophs was found to be even higher among harmful algal bloom species, at almost 74% (*n* = 27) [63]. Hence, the ability to synthesize this vital molecule might confer an ecological advantage to marine protists, rather than scavenging exogenous thiamine. Previous studies have indicated that *P. parvum* is a thiamine auxotroph [64] and that prymnesiophytes in general tend to require thiamine for growth [56], [63]. Our dataset indicates a difference between the two *Chrysochromulina* species and *P. parvum* and *P. antarctica* in the number of enzymes for thiamine synthesis present in their transcriptome, implying that these species may also differ in their ability to make thiamine. Based on the genes that are present in the transcriptomes, it would seem that *P. parvum* and *P. antarctica* are least likely to be able to synthesize thiamine while *C. brevifilum* and *C. ericina* may be able to synthesize the vitamin, either *de novo*, or from an intermediate in the pathway. Past studies have shown that in some species, the need for exogenous thiamine was alleviated when either the thiazole or pyrimidine moiety was added to the growth medium [65]. This might provide a species with some flexibility competing against other organisms that specifically require thiamine for growth. It is also possible that the ThiD, ThiE, ThiDE, ThiF and TPK enzymes that are present in the two *Chrysochromulina* spp. are remnants of the thiamine pathway and do not represent a functional thiamine biosynthesis pathway. Some of these genes have also been found in *Ostreococcus tauri* and *Micromonas pusilla* CCMP1545, both of which are thiamine auxotrophs [62], [66].

Biotin is a cofactor for carboxylase enzymes that are used in fatty acid synthesis, and thus is required across all domains of life. All the haptophytes surveyed in a previous study did not require biotin [56] but it was not an exhaustive survey. None of our four target species contained all three biotin synthesis genes found in *C. reinhardtii*, *T. pseudonanna* and *C. merolae*, three species capable of biotin synthesis. It is, of course, still possible that a functional biosynthetic pathway is present in our target prymnesiophytes due to the presence of yet-unidentified enzymes.

So far, only prokaryotes have been shown to synthesize cobalamin, but many protists require cobalamin for the synthesis of amino acids and deoxyriboses, and for C1 metabolism. Examples of enzymes that require cobalamin include methionine synthase (METH) and methylmalonyl coA mutase (MCM). Previous studies have shown *P. parvum* to have a specific and non-replaceable requirement for cobalamin in its growth media [67], [68], and *P. globosa*, which is a congener of *P. antarctica*, also requires exogenous cobalamin [63]. The lack of all but one or two genes in the cobalamin biosynthetic pathway and the necessity of exogenous cobalamin in the growth media of most microalgae strongly indicate that all four of our study species are unable to synthesize cobalamin and are dependent on external cobalamin. Additionally, only METH was present in our datasets, and not METE, the cobalamin-independent form of methionine synthase. The latter is strongly correlated with cobalamin independence [69]. We also found putative MCM genes in all four transcriptomes, further evidence for cobalamin dependence among our four target organisms.

Macronutrients such as nitrogen and phosphorus have long been known to be important factors structuring species composition and distribution in the ocean, but in recent years micronutrients such as vitamins have been found to also play an important role [56], [63], [66]. Our comparative transcriptome analysis of four prymnesiophytes has revealed potential differences in the ability of these closely related species to synthesize some B-vitamins, perhaps indicating unique metabolic abilities or dependences that might explain differences in their autecologies.

### Polyketide synthase

Polyketide synthase genes are thought to be involved in the synthesis of at least some of the toxins that have been found in *P. parvum* and *C. polylepsis* [70], [71]. Two of the toxins produced by *P. parvum* that have been isolated to date, prym1 and prym2 [70], [72], [73] are ladder-like polycyclic ethers that resemble other algal toxins produced by Type I PKS genes such as brevitoxin and okadaic acid, the latter compounds produced by marine dinoflagellates [74], [75].

Type I PKS are modular, multi-domain proteins that are similar to proteins involved in fatty acid synthesis (FAS). These proteins sequentially add acyl units onto a growing carbon chain via a condensation reaction. The following three domains are required for the synthesis of polyketide molecules: ketosynthase (KS), acyltransferase (AT) and acyl-carrier protein (ACP). Additional domains encode ketoreductase (KR), dehydratase (DH) and enoyl reductase (ER) proteins, which catalyze the reduction of the initial 2-, 3- and 4-carbon skeletons. The thioesterase (TE) domain releases the polyketide molecule from its attachment site when the final chain length has been achieved [70].

In general, these domains are organized into modules, with each module containing the domains that are required for one round of chain elongation and modification [70]. However, PKS sequences have been found in *K. brevis* that contain only one or two catalytic domains [75], similar to some of the sequences in our dataset. A previous study in *C. polylepsis* found KS, KR and AT domains in their EST dataset, but no information was provided on how these domains were organized, likely due to the short average sequence length (∼600 bp) [72]. As such, there is insufficient information to ascertain what a ‘typical’ PKS gene might look like in a prymnesiophyte. Even within the transcriptome of a single species, i.e. *C. brevifilum*, the putative PKS sequences were of different lengths and had different numbers and organization of domains (Fig. 3). Thus, they may be responsible for synthesizing polyketide molecules of different lengths and configurations.

Our results also indicated the existence of two different KS gene families within our prymnesiophyte datasets, one comprising prymnesiophyte-specific sequences and one containing sequences from diverse bacterial and protistan species (Fig. 2). All of the *P. parvum* KS sequences clustered with the latter clade. *P. parvum* was grown axenically in our study, thus the sequences could not have been derived from a bacterium. While the mixed bacterial/protistan clade itself is not well-supported, the subclade containing the *P. parvum*, *P. antarctica* (except one) and *E. huxleyi* sequences was a well-supported clade in our dataset. A previously sequenced *P. parvum* PKS sequence from an EST library [76] was also found within this mixed bacterial/protistan PKS cluster. It is unknown if the toxigenic *C. polylepsis* KS sequences would group with the *P. parvum* and *P. antarctica* sequences or with the *C. brevifilum* sequences, but to our knowledge the *C. polylepsis* sequences are not in public databases.

The prymnesiophyte-specific clade was more closely related to the *Ostreococcus* sequences than to the dinoflagellate-specific clade, a finding similar to a previous study [77], and may suggest a common origin for green algal and prymnesiophyte PKS distinct from that of the dinoflagellates. It may be significant that all of the PKS sequences that clustered with the haptophyte-specific clade contained multiple domains whereas the sequences in the mixed bacterial/protistan clade only contained the KS domain. However, it is important to be cautious when interpreting these results because this and other PKS trees tend to have large sequence divergences and lack a suitable outgroup, which results in poorly supported branching order.

### Analysis of KOG relative abundances reveals interesting clustering patterns

The functional annotations of the four prymnesiophytes using the KOG database did not differ markedly, presumably because they share similar core functions (Fig. 4). This similarity may be a consequence of the close phylogenetic relationship among these four species, or because they share similar physiologies or nutritional modes, factors which are not mutually exclusive. Three of the four prymnesiophytes examined in this study exhibit phagotrophic behavior but one, *P. antarctica*, is so far not known to be mixotrophic [78]. Nonetheless, there were no clear differences in the KOG functions between the three known mixotrophs and the non-mixotrophic *P. antarctica* in our study (Fig. 4). However, when we included KOG data from other protistan species in a non-metric multidimensional scaling (NMDS) analysis and a principal components analysis (PCA), some interesting patterns emerged (Figs. 5, 6). Our subsequent statistical analysis therefore took into account the effects of both trophic mode and phylogenetic grouping.

Phylogenetic identity appeared to be a significant determinant of the species clusters on the NMDS plot (Fig. 5). For example, alveolate taxa (dinoflagellates and a ciliate) clustered separately from all other species, presumably indicating a strong phylogenetic signal in their transcriptomes. Dinoflagellates generally have large genomes, and a lot of genes appear to be constitutively expressed and modified post-translationally [79]. This tendency might result in a greater variety of transcribed genes and hence, larger variations in their transcriptomes and in their KOG distribution patterns, and explain why these organisms occupy a location far away from other species on the NMDS plot, as well as being relatively spread out from each other compared to other phylogenetic groups.

Other apparently phylogenetic-based groupings on the NMDS plot included the diatoms which all clustered close to on another, and the chlorophytes with one notable exception. The outlier from this cluster,*C. reinhardtii*, showed greater similarity to heterotrophic species than to its congener, *Chlamydomonas* sp. CCMP681. *Chlamydomonas* sp. CCMP681 [80] was isolated from the Southern Ocean near Antarctica while *C. reinhardtii* is usually found in freshwater ecosystems and in soil [81]. We speculate that the substantial distance between these congeners on the NMDS plot could be due to differences in physiological adaptations to very different habitats. Interestingly, the two *Ochromonas* species were also situated some distance from each other on the NMDS plot. *Ochromonas* clone CCMP1899 was isolated from the Ross Sea, Antarctica, while clone BG-1 is a freshwater isolate from a botanical garden in Malaysia.

Another interesting pattern observed in the NMDS analysis was the tendency for organisms to cluster based on similar nutritional modes but distant phylogenetic relationships. Heterotrophic taxa, including the water molds (oomycetes), a slime mold (mycetozoa), a choanoflagellate and a heterotrophic chrysomonad all clustered away from those taxa possessing phototrophic ability (including the kleptoplastidic ciliate, *Mesodinium pulex*). This pattern is perhaps not unexpected because these heterotrophic taxa would presumably lack the photosynthetic machinery possessed by phototrophic protists, but it is interesting that the broad grouping of the transcriptomes of these heterotrophs appear to reflect their nutritional mode.

*Aureococcus anophagefferens* and *Chlamydomonas reinhardtii* are labeled as phototrophs in the NMDS plot, yet these taxa occurred relatively close to the heterotrophic protists. As noted above, the habitat for *C. reinhardtii* is quite different than for most of the photrophic protists examined in this study. *A. anophagefferens* has strong osmotrophic capabilities [82], [83], which may explain its proximity to the other heterotrophs on the NMDS plot, albeit at lesser proximity than *C. reinhardtii* to the heterotrophs.

The non-alveolate mixotrophs in our dataset (chrysophytes and prymnesiophytes, including three of the four species examined in this study) formed a cluster on the NMDS plot that occupied a central space between the alveolates on the left side of the plot, chlorophytes and diatoms above, and heterotrophs to the right (Fig. 5A). Our fourth prymnesiophyte, *P. antarctica*, also clustered with these species. Their intermediate position on the plot between purely (or predominantly) photosynthetic organisms and exclusively heterotrophic species may reflect the mixed nutritional mode that is characteristic of these organisms. Phagotrophic algae possess the cellular machinery that allows them to carry out photosynthesis, therefore their KOG distribution might be expected to exhibit a fair amount of similarity with phototrophic protists. It is interesting that while a comparison of four closely related species did not reveal large differences in KOG functions, comparing these four species with a larger set of protistan taxa resulted in distinct clusters based on phylogeny, or nutritional mode, or both, despite the fact that only a small fraction of the transcriptomes of these organisms could be assigned KOG annotations at this time. One might expect that physiological differences between heterotrophs, phototrophs and mixotrophs could be explained by the presence or absence of particular pathways (e.g. photosynthetic pathways), but the distribution of annotated genes within certain KOG functions were also different. Our data provide some good starting points for probing more in-depth differences among protists with different nutritional modes. For instance, it might be expected that an in-depth analysis of KOG category G (carbohydrate transport and metabolism) might unveil a greater diversity of isoenzymes to process and digest different sugars synthesized by prey. In this regard, we also observed differences in the KOG category H (coenzyme transport and metabolism), which is not that surprising as phototrophs and heterotrophs would likely have differences in this category because prey biomass might be able to supply some of these necessary molecules for enzymatic reactions.

Our data and analysis have demonstrated the utility of transcriptomic data for analyzing functional and physiological capabilities of closely-related or nutritionally similar protists. Despite the present paucity of reference databases that presently allow only a small fraction of the peptides in this study to be annotated, we were nonetheless able to gain insight by comparing the four transcriptomes to each other, and to other transcriptomes that were available in public databases. The ability to more fully annotate these datasets will add significantly to the depth of future analyses, by enabling a fuller elucidation of pathways and functions that are shared or novel among the species. In this study, we were able to show that four prymnesiophytes share a set of core genes that mostly comprise the essential metabolic and cellular pathways in the cell. We also found evidence to suggest that investigations into functional and perhaps, by extension, ecological differences between closely related species should be focused on “secondary” pathways such as vitamin biosynthesis or secondary metabolic pathways. Finally, our data indicated that the nutritional mode of a species, as well as its phylogeny, can influence the proportion of its genome that is devoted to specific KOG functions.

## Supporting Information

File S1 **Contains Tables S1–S4 and Figure A. Table S1**. Number of contigs containing rRNAs and tRNAs in each transcriptome. **Table S2. Predicted proteins related to cobalamin biosynthesis.** METH: B12-dependent methionine synthase; MCM: methylmalonyl-CoA mutase; CobB: cobyrinic acid a,c-diamine synthase; CobNST: CobN subunit of cobaltochelatase; CobW: protein putatively involved in cobalamin biosynthesis but its specific catalytic role is unclear. **Table S3. Proteins containing polyketide synthase ketosynthase (KS) domains.** **Table S4. Results of the non-parametric Krustal-Wallis tests for each KOG function.** The influence of phylogeny and trophic mode was tested independently with a non-parametric Krustal-Walis test followed by a Steel-Fligner test if significant differences were observed. All calculations done with XLSTAT (v.2013.06.04, Adinsoft TM) with an alpha of 0.01. Two data sets were used in this statistical analysis: (1) a dataset with most of the species present in Figure 4 but for the Mycetozoa *D. purpureaum*, the Choanoflagellate *M. brevicollis* and the Rhodophyte *C. merolae* due to statistical reasons; and (2) a reduced dataset considering only the Stramenopiles and Prymnesiophyta. Abreviations as follows: NS, not significant; YES, significant difference detected. **Figure A. Key components of the thiamine biosynthesis pathway.** Colored squares represent presence in *Prymnesium parvum* (brown), *Chrysochromulina brevifilum* (red), *Chrysochromulina ericina* (blue), and *Phaeocystis antarctica* (green). In some organisms, the functionalities of THID and ThiE are combined into a single enzyme, such as ThiDE. Abbreviations: HMP-P, 4-amino-2-methyl-5-hydroxymethylpyrimidine phosphate; HMP-PP, 4-amino-2-methyl-5-hydroxymethylpyrimidine pyrophosphate; HET-P, hydroxyethylthiazole phosphate; DXP, 1-deoxy-D-xylulose 5-phosphate.
(DOCX)
