# Supplementary material for: Comparative Transcriptome Analysis of Four Prymnesiophyte Algae
Source: PLoS One. 2014 Jun 13;9(6):e97801. doi: 10.1371/journal.pone.0097801 (PMC4057078; doi:10.1371/journal.pone.0097801)
Supplement: File S1 — Contains Tables S1–S4 and Figure A. Table S1. Number of contigs containing rRNAs and tRNAs in each transcriptome. Table S2. Predicted proteins related to cobalamin biosynthesis. METH: B12-dependent methionine synthase; MCM: methylmalonyl-CoA mutase; CobB: cobyrinic acid a,c-diamine synthase; CobNST: CobN subunit of cobaltochelatase; CobW: protein putatively involved in cobalamin biosynthesis but its specific catalytic role is unclear. Table S3. Proteins containing polyketide synthase ketosynthase (KS) domains. Table S4. Results of the non-parametric Krustal-Wallis tests for each KOG function. The influence of phylogeny and trophic mode was tested independently with a non-parametric Krustal-Walis test followed by a Steel-Fligner test if significant differences were observed. All calculations done with XLSTAT (v.2013.06.04, Adinsoft TM) with an alpha of 0.01. Two data sets were used in this statistical analysis: (1) a dataset with most of the species present in Figure 4 but for the Mycetozoa D. purpureaum, the Choanoflagellate M. brevicollis and the Rhodophyte C. merolae due to statistical reasons; and (2) a reduced dataset considering only the Stramenopiles and Prymnesiophyta. Abreviations as follows: NS, not significant; YES, significant difference detected. Figure A. Key components of the thiamine biosynthesis pathway. Colored squares represent presence in Prymnesium parvum (brown), Chrysochromulina brevifilum (red), Chrysochromulina ericina (blue), and Phaeocystis antarctica (green). In some organisms, the functionalities of THID and ThiE are combined into a single enzyme, such as ThiDE. Abbreviations: HMP-P, 4-amino-2-methyl-5-hydroxymethylpyrimidine phosphate; HMP-PP, 4-amino-2-methyl-5-hydroxymethylpyrimidine pyrophosphate; HET-P, hydroxyethylthiazole phosphate; DXP, 1-deoxy-D-xylulose 5-phosphate. (DOCX) [file pone.0097801.s001.docx]

**Table S1**. Number of contigs containing rRNAs and tRNAs in each transcriptome.

| Species | rRNAs | tRNAs |
| --- | --- | --- |
| *P. parvum* | 2 | 3 |
| *C. brevifilum* | 8 | 0 |
| *C. ericina* | 17 | 13 |
| *P. antarctica* | 7 | 1 |

|  |  |  |  |
| --- | --- | --- | --- |

**Table S2. Predicted proteins related to cobalamin biosynthesis.** METH: B12-dependent methionine synthase; MCM: methylmalonyl-CoA mutase; CobB: cobyrinic acid a,c-diamine synthase; CobNST: CobN subunit of cobaltochelatase; CobW: protein putatively involved in cobalamin biosynthesis but its specific catalytic role is unclear.

| **ORF** | **Annotation** |
| --- | --- |
| ***P. parvum***  6621_1  5553_1  19779_1, 17579_1 | METH  MCM  CobW |
| ***C. brevifilum***  63818_1  109570_1  26401_1, 110009_1, 78490_1  13986_1 | METH  MCM  CobW  CobB |
| ***C. ericina***  13279_1, 32542_1  29808_1  35493_1, 40785_1, 50658_1, 16219_1, 10713_1, 45973_1, 26167_1, 3438_1, 2169_1, 38987_1  39377_1 | METH  MCM  CobW  CobNST |
| ***P. antarctica***  27826_1, 46214_1, 11053_1  38804_1, 38597_1, 46771_1  24567_1, 7519_1, 17091_1, 24039_1, 41170_1, 28986_1  35735_1 | METH  MCM  CobW  CobNST |

**Table S3. Proteins containing polyketide synthase ketosynthase (KS) domains.**

| **Protein ID** | **Species** | **PKS domains** |
| --- | --- | --- |
| 18839 | *P. parvum* | KS |
| 2015 | *P. parvum* | KS |
| 26981 | *P. parvum* | KS |
| 30494 | *P. parvum* | KS |
| 7797 | *P. parvum* | KS |
| 106225 | *C. brevifilum* | KS, KR, PP |
| 106729 | *C. brevifilum* | KS, DH, KR, PP |
| 107078 | *C. brevifilum* | KS, PP, KR |
| 107100 | *C. brevifilum* | KS, PP |
| 4093 | *P. antarctica* | KS, PP |
| 15245 | *P. antarctica* | KS |
| 23625 | *P. antarctica* | KS |
| 4182 | *P. antarctica* | KS |
| 49699 | *P. antarctica* | KS |
| 54160 | *P. antarctica* | KS |

**Table S4. Results of the non-parametric Krustal-Wallis tests for each KOG function.** The influence of phylogeny and trophic mode was tested independently with a non-parametric Krustal-Walis test followed by a Steel-Fligner test if significant differences were observed. All calculations done with XLSTAT (v.2013.06.04, Adinsoft TM) with an alpha of 0.01. Two data sets were used in this statistical analysis: (1) a dataset with most of the species present in Figure 4 but for the Mycetozoa *D. purpureaum*, the Choanoflagellate *M. brevicollis* and the Rhodophyte *C. merolae* due to statistical reasons; and (2) a reduced dataset considering only the Stramenopiles and Prymnesiophyta. Abreviations as follows: NS, not significant; YES, significant difference detected.

| Original dataset | | | Reduced dataset | | | |
| --- | --- | --- | --- | --- | --- | --- |
| KOG | Phyla | Type | KOG | Phyla | Type |  |
| A | NS | NS | A | NS | NS |  |
| **B** | **YES** | NS | B | NS | NS |  |
| **C** | **YES** | NS | C | NS | NS |  |
| **D** | NS | **YES** | D | NS | **YES** |  |
| E | NS | NS | E | NS | NS |  |
| F | NS | NS | F | NS | NS |  |
| **G** | NS | **YES** | G | NS | **YES** |  |
| **H** | NS | **YES** | H | NS | **YES** |  |
| **I** | **YES** | NS | I | NS | NS |  |
| J | NS | NS | J | NS | NS |  |
| **K** | **YES** | **YES** | K | NS | **YES** |  |
| **L** | **YES** | **YES** | L | NS | NS |  |
| **M** | **YES** | NS | M | NS | NS |  |
| N | NS | NS | N | NS | NS |  |
| **O** | **YES** | NS | O | NS | NS |  |
| P | NS | NS | P | NS | NS |  |
| Q | NS | NS | Q | NS | NS |  |
| R | NS | NS | R | NS | NS |  |
| T | NS | NS | T | NS | NS |  |
| **U** | **YES** | NS | U | NS | NS |  |
| V | NS | NS | V | NS | NS |  |
| W | NS | NS | W | NS | NS |  |
| **Y** | **YES** | NS | Y | NS | NS |  |
| **Z** | **NS** | **YES** | **Z** | **NS** | **NS** |  |

**
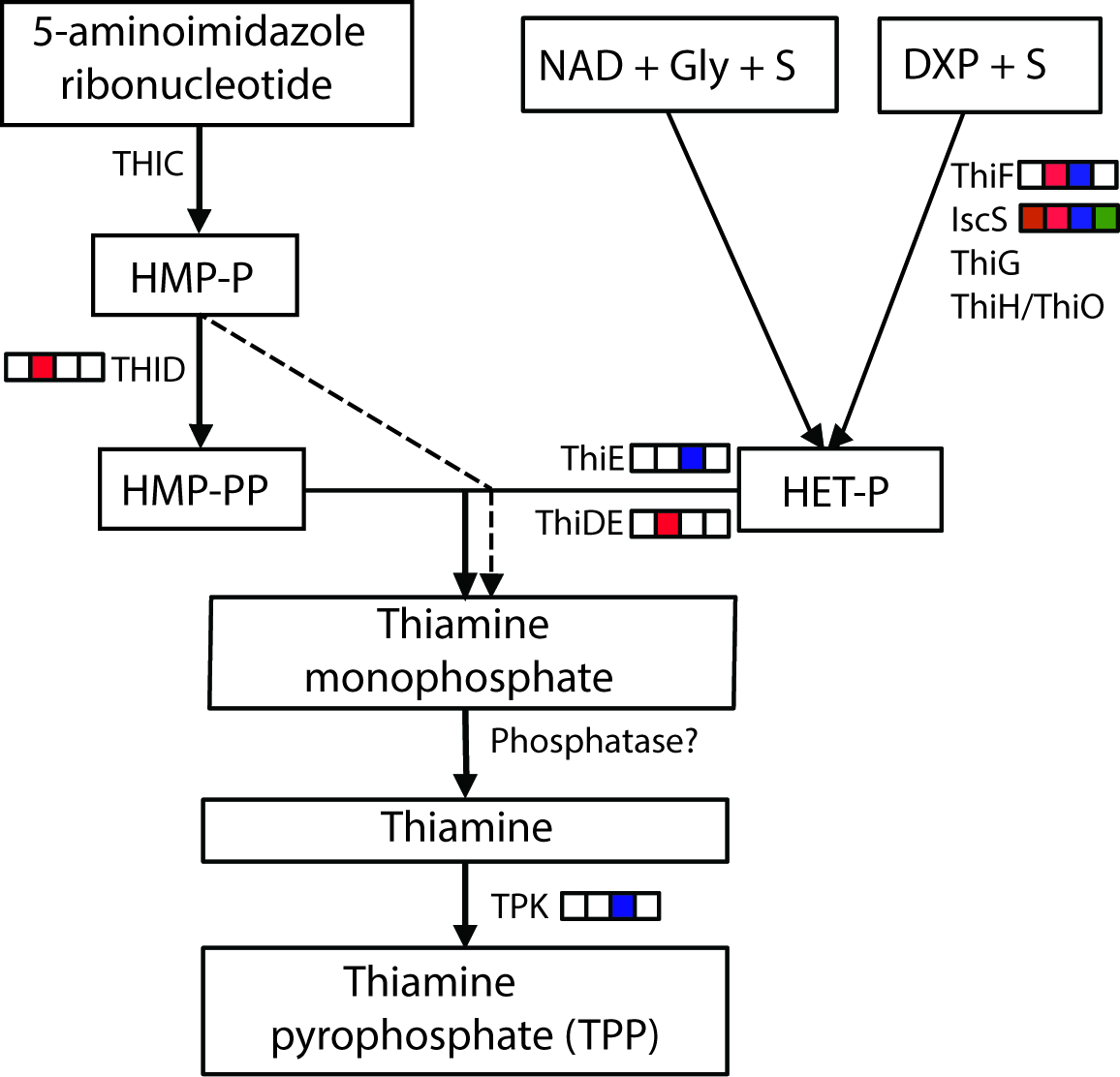
**

**Figure A. Key components of the thiamine biosynthesis pathway.** Colored squares represent presence in *Prymnesium parvum* (brown), *Chrysochromulina brevifilum* (red), *Chrysochromulina ericina* (blue), and *Phaeocystis antarctica* (green). In some organisms, the functionalities of THID and ThiE are combined into a single enzyme, such as ThiDE. Abbreviations: HMP-P, 4-amino-2-methyl-5-hydroxymethylpyrimidine phosphate; HMP-PP, 4-amino-2-methyl-5-hydroxymethylpyrimidine pyrophosphate; HET-P, hydroxyethylthiazole phosphate; DXP, 1-deoxy-D-xylulose 5-phosphate.
